# Cardiorespiratory fitness status of elite handball referees in Hungary

**DOI:** 10.1371/journal.pone.0270999

**Published:** 2022-07-07

**Authors:** Máté Babity, Márk Zámodics, Bálint K. Lakatos, Réka Rákóczi, Albert König, Anna Menyhárt-Hetényi, Alexandra Fábián, Anna Kiss, Márton Tokodi, Attila Kovács, Hajnalka Vágó, Béla Merkely, Orsolya Kiss

**Affiliations:** 1 Heart and Vascular Center, Semmelweis University, Budapest, Hungary; 2 Department of Sports Medicine, Semmelweis University, Budapest, Hungary; Universidade Estadual Paulista Julio de Mesquita Filho - Campus de Bauru, BRAZIL

## Abstract

In various team sports, such as handball, referees work on the court by continuously moving with the players. Therefore, their physical fitness also has an impact on their reaction time, which could affect their professional decisions. The cardiorespiratory fitness status of healthy Hungarian elite handball referees was examined via body composition analysis and vita maxima cardiopulmonary exercise testing with lactate measurements. One hundred referees were examined (age: 29.0 ± 7.9 years; male: 64.0%; training: 4.3 ± 2.0 hours/week; ratio of former elite handball players: 39.0%; 51.0% first and 49.0% second division referees of the Hungarian National Handball Leagues). A resting heart rate (HR) of 79.0 ± 12.6 BPM was measured. On the basis of the body composition analysis the fat-free mass index proved to be 19.9 ± 2.6 kg/m^2^. The referees achieved a maximal oxygen uptake (V̇O_2max_) of 44.6 ± 6.1 ml/kg/min, with a maximal HR of 187.2 ± 11.1 BPM (which was 98.1 ± 4.6% of their calculated maximal HR) and a peak lactate of 9.2 ± 3.2 mmol/l at 557.1 ± 168.3 sec on our continuous speed, increasing slope treadmill protocol. Second division referees were younger, on a weekly average they trained more, achieved higher treadmill exercise time (respectively, 463.8 ± 131.9 vs 658.4 ± 143.9 sec, p < 0.001) and anaerobic threshold time (respectively, 265.8 ± 100.9 vs 348.2 ± 117.1 sec, p < 0.001), while the two different divisional referees had similar V̇O_2max_ values. Regarding our physical fitness measurements, huge individual differences were observed between the referees (exercise time range: 259.0–939.0 sec, V̇O_2max_ range: 25.3–62.4 ml/kg/min). Since it can affect their performance as referees, individual training planning, regular physical fitness measurements, and strict selection methods are suggested.

## Introduction

In various team sports, such as handball, football and basketball, referees work on the field or the court, continuously moving with the game and the players. Previous results described the prevalence of cardiovascular diseases and risk factors in elite handball referees [1]. These referees are expected to have a good aerobic power (oxygen uptake, V̇O_2_). At greater loads, inappropriate physical fitness could negatively influence cognitive functions, which is essential to referee in a fair way, always according to the official rules and regulations of the refereed game [2, 3]. In addition to physical load, the inseparable mental stress also exerts a great impact on the referees’ decision making [4].

Competitive handball is a high-intensity mixed team sport [5]. The rules of the game have been established by the International Handball Federation and are regularly revised [6]. The games are played indoors on a 20 m x 40 m court, with 7 players from each team (in the basic lineup, 6 field players and 1 goalkeeper), allowing for multiple substitutions during the 60 minutes of the game (divided to 2 halves). During the game, the players usually run around 4–5 km, and perform multiple high-intensity runs and sprints, as well as faults, as handball is a contact sport [7–9]. Two referees are nominated for each game, both of them are moving with the offensive and defensive actions of both teams [6].

In Hungary, handball referees must pass multiple tests annually; these include shuttle running, 12-minute continuous running, and rule- and video tests. The minimum requirements are determined by the Hungarian Handball Federation (HHF). These physical tests are used to measure the fitness status of the referees roughly, since the tests are standardized and are easy to perform regularly, only allowing for the estimation of the maximal oxygen uptake (V̇O_2max_) [10]. With cardiopulmonary exercise testing (CPET), we are able to measure the maximal physiological levels of vital parameters, such as exact V̇O_2_, anaerobic thresholds, and the kinetics of heart rate (HR), V̇O_2_, and lactate levels during exercise [11]. However, these values can have a great impact on decision making, they alone cannot define the performance of the referees during the games, because previous experience and mental skills are also required [12]. Existing literature data suggest that the decision-making of the referees is not only determined by the time spent in the anaerobic phase, but rather by the rating of perceived exertion (RPE), which could be lowered by regular training [13].

The aim of this study was to determine the fitness level of top Hungarian handball referees to examine personal and gender-based differences, as well as the differences between referees who participate in the first vs the second division of the Hungarian National Handball Leagues (HNHL).

## Materials and methods

### Participants

In this study, 100 asymptomatic elite referees of the HHF of both sexes, aged 18 and above, were examined. Any symptoms of internal-, cardiovascular-, or musculoskeletal diseases counted as exclusion criteria. All the examined referees were Caucasian. The referees were selected in cooperation with the Referee Subcommittee of the HHF to include the 100 best Hungarian handball referees in this study. Prior to the study, all participants gave written informed consent to the examinations and the research purposes after having been provided verbal information and answers to all the arising questions. The Medical Research Council of Hungary approved (13687-1/2011-EKU) the study according to the Ethical Guidelines of the Helsinki Declaration.

### Procedures

All measurements were performed at least 12 hours after the last training session or refereeing [14]. To ensure similar conditions for all referees, measurements took place in the morning hours during the playoff period of the season, in an air-conditioned laboratory with constant temperature and humidity. Subjects with symptoms or who were suspended from regular physical activity in the last 6 months were not invited to take part in the examinations. Prior to the CPET examinations, all participants went through routine cardiology examinations. A detailed questionnaire was implemented to record detailed data of sports activity and referee career. Body-composition analyses were carried out with bioelectrical impedance measurements (Bodystat 1500 MDD, Bodystat Ltd, UK; InBody 770, InBody Co. Ltd, South Korea) [15, 16]. Body mass index (BMI) was calculated as *BMI [kg/m*^*2*^*] = body weight [kg] / body height*^*2*^ *[m*^*2*^*]*, and fat-free mass index (FFMI) as *FFMI [kg/m*^*2*^*] = body fat-free weight [kg] / body height*^*2*^ *[m*^*2*^*]* [17]. Cardiopulmonary exercise testing was implemented on a treadmill ergometer (T-2100, GE Healthcare, Finland) with an incremental protocol starting with a 1-minute flat walk of 6 km/h, followed by continuous 10 km/h uphill running with an increasing slope of 1.0% every minute until exhaustion. After stopping running, measurements were continued during a 1-minute 4 km/h walk and a further 4-minute rest [18]. Breath-by-breath gas analysis was carried out with an automated cardiopulmonary exercise system (Respiratory Ergostik, Geratherm, Germany; Blue Cherry V1.3.3.1, Geratherm, Germany) [19]. The reference values for non-athletes were integrated to the system by the manufacturer considering sex, age, height, and weight. Continuous electrocardiography (ECG) and HR monitorization were carried out (CAM-14 module, GE Healthcare, Finland; CardioSoft V6.73, GE Healthcare, Finland), the estimated maximal HR was calculated as *220-age* [5]. The HR values were calculated as the average of 10-second measurements. Blood lactate levels were measured from fingertip capillary blood drops at rest, during the exercise in every second minute, at maximal load, and in the fifth minute of the cool-down (Laktate Scout 4+, EKF Diagnostik, Germany). Anaerobic threshold was determined based on the lactate levels and the kinetics of the recorded Wasserman graphs [20]. All examinations and data collection were supervised by a cardiology specialist.

### Statistical analysis

Descriptive statistical values are shown as mean ± SD (range: minimum-maximum, 95% confidence interval (CI_95%_): lower-limit—upper-limit), comprehensive statistical analysis was carried out for continuous data with Wilcoxon rank sum test (Mann–Whitney U test) or two-tailed unpaired Student’s t-probe with equal or unequal variance form, depending on the normality of the valuables; and with Chi-square test for discrete data. The normality of the valuables was tested with the Shapiro–Wilk test, the homogeneity of variances was tested with F-test. The effect size analyses were carried out using Cohen’s d formula, Glass rank-biserial correlation or Phi coefficient in accordance with the statistical tests performed [21]. Effect sizes were considered as “small”, “medium” and “large” if the effect size analyses were 0.2–0.49, 0.5–0.79 and ≥0.8, respectively) [22]. Correlation analysis was performed via the Pearson correlation. Statistical significance was determined as p < 0.05. Statistical analyses were performed using a dedicated software (Microsoft Excel, Microsoft Corporation, USA; Real Statistics Resource Pack software (Release 7.6), Copyright (2013–2021) Charles Zaiontz) [23]. All missing data were proved to be missing completely at random, thereby available-case analysis was carried out for the statistic evaluation.

## Results

### Study population

We examined 100 Hungarian elite handball referees (age: 29.0 ± 7.9 years, range: 18–46 years, CI_95%_: 27.4–30.6 years; male: 64.0%). On average, they trained 4.3 ± 2.0 hours/week (range: 1–11.5 hours/week, CI_95%_: 3.9–4.7 hours/week), 39.0% were former elite handball players. At the time of the examinations, 51.0% were first and 49.0% were second division referees of the HNHL. A 16.0% of the referees also officiated games for either the International Handball Federation or the European Handball Federation. Data according to the level of refereeing can be found in Table 1.

**Table 1:** Basic parameters of Hungarian elite handball referees according to the division of refereeing.

	All referees mean ± SD (95% CI)	First division referees mean ± SD (95% CI)	Second division referees mean ± SD (95% CI)	First vs Second division referees	
				p-value	Effect size
Participant [N (%)]	100 (100.0)	51 (51.0)	49 (49.0)	-	-
Male [N (%)]	64 (64.0)	39 (76.5)	25 (51.0)	*0*.*008*	0.265^#^
Age (year)	29.0 ± 7.9 (27.4–30.6)	33.0 ± 8.1 (30.7–35.2)	24.8 ± 5.2 (23.3–26.3)	*< 0*.*001*	0.571^##^
Training (hours/week)	4.3 ± 2.0 (3.9–4.7)	3.8 ± 2.0 (3.3–4.4)	4.8 ± 2.0 (4.2–5.4)	*0*.*006*	0.315^#^
Height (cm)	178.0 ± 8.1 (176.4–179.7)	179.6 ± 8.9 (177.1–182.1)	176.4 ± 7.0 (174.4–178.5)	0.055	-
Weight (kg)	78.0 ± 13.3 (75.4–80.7)	81.5 ± 13.6 (77.7–85.4)	74.3 ± 12.0 (70.9–77.8)	*0*.*005*	0.323^#^
BMI (kg/m^2^)	24.5 ± 2.7 (23.9–25.0)	25.1 ± 2.3 (24.4–25.7)	23.8 ± 3.0 (23.0–24.7)	*0*.*016*	0.477^#^
Body fat (%)	18.7 ± 6.6 (17.3–20.0)	16.4 ± 5.4 (14.9–17.9)	21.0 ± 6.9 (19.0–23.0)	*< 0*.*001*	0.694^##^
FFMI (kg/m^2^)	19.9 ± 2.6 (19.4–20.4)	21.0 ± 2.1 (20.4–21.5)	18.8 ± 2.7 (18.0–19.5)	*< 0*.*001*	0.245^#^
Former elite player [N(%)]	39 (39.0)	23 (45.1)	16 (32.7)	0.202	-

Abbreviations: BMI, Body Mass Index; FFMI, Fat-free Mass Index, 95% CI, 95% confidence interval. Statistical analysis was carried out between the First and Second division referees.

^#^ small effect size,

^##^ medium effect size.

### Resting examinations

None of the examined referees reported any cardiovascular symptoms or previous cardiovascular diseases, except for known hypertension in 4.0% and ablated AV-reentry tachycardia in 1 case. During the resting examinations, no serious cardiovascular diseases were found. Physiological, sports-related ECG changes were found in 55.0%. The results of the body composition analysis are shown in Table 1.

### Cardiopulmonary exercise testing

On our running protocol, the average running time was 557.1 ± 168.3 sec for all referees (range: 259.0–939.0 sec, CI_95%_: 423.4–590.9 sec), with a maximal HR of 187.2 ± 11.1 BPM (range: 157–210 BPM, CI_95%_: 185.0–189.4 BPM), which was 98.1 ± 4.6% of their calculated maximal values. The referees achieved a maximal ventilation of 128.1 ± 29.7 l/min (range: 69.0–207.0 l/min, CI_95%_: 122.2–134.1 l/min), with V̇O_2max_ of 44.6 ± 6.1 ml/kg/min (range: 25.3–62.4 ml/kg/min, CI_95%_: 43.4–45.8 ml/kg/min) and a peak lactate level of 9.2 ± 3.2 mmol/l (range: 3.1–18.9 mmol/l, CI_95%_: 8.5–9.8 mmol/l). On average, the referees achieved their anaerobic threshold at 56.1 ± 15.8% of the exercise time, with 91.5 ± 6.5% of their maximal HR and with 86.5 ± 8.2% of their V̇O_2max_. Data are shown in Table 2. There was no significant difference in the exercise time between males and females (respectively, 575.0 ± 168.2 vs. 526.4 ± 166.4 sec; p = 0.17), while V̇O_2max_ proved to be higher in male referees (respectively, 47.0 ± 5.7 vs. 40.4 ± 4.3 ml/kg/min; p < 0.001, effect size: 0.961 (large)).

**Table 2:** Cardiopulmonary exercise testing results of Hungarian elite handball referees according to the division of refereeing.

	All referees mean ± SD (95% CI)	First division referees mean ± SD (95% CI)	Second division referees mean ± SD (95% CI)	First vs Second division referees	
				p-value	Effect size
Resting HR (BPM)	79.0 ± 12.6 (76.5–81.5)	78.7 ± 11.1 (75.6–81.9)	79.3 ± 14.1 (75.2–83.4)	0.745	-
Exercise testing time (sec)	557.1 ± 168.3 (523.4–590.9)	463.8 ± 131.9 (426.7–500.9)	658.4 ± 143.9 (616.1–700.6)	*< 0*.*001*	0.688^##^
Maximal HR (BPM)	187.2 ± 11.1 (185.0–189.4)	183.3 ± 15.9 (178.8–187.7)	190.0 ± 10.2 (187.0–192.9)	*0*.*012*	0.294^#^
Peak lactate level (mmol/l)	9.2 ± 3.2 (8.5–9.8)	9.0 ± 3.6 (8.0–10.0)	9.5 ± 2.6 (8.7–10.3)	0.421	-
V̇O_2max_ (ml/kg/min)	44.6 ± 6.1 (43.4–4.8)	45.1 ± 5.6 (43.6–46.7)	44.0 ± 6.7 (42.0–46.0)	0.291	-
V̇O_2max_ compared to non-athlete reference(%)	124.9 ± 14.3 (122.1–127.8)	126.5 ± 13.6 (122.6–130.3)	123.3 ± 15.0 (118.9–127.7)	0.275	-
Maximal ventilation (l/min)	128.1 ± 29.7 (122.2–134.1)	134.0 ± 28.1 (126.1–141.9)	121.8 ± 30.3 (112.9–130.7)	*0*.*016*	0.264^#^
Maximal ventilation compared to non-athlete reference (%)	119.6 ± 17.2 (116.2–123.1)	120.9 ± 15.0 (116.7–125.2)	118.2 ± 19.4 (112.5–123.9)	0.430	-
AT time (sec)	306.2 ± 116.2 (282.6–329.7)	265.8 ± 100.9 (234.0–278.8)	348.2 ± 117.1 (313.8–382.6)	*< 0*.*001*	0.437^#^
AT time compared with CPET time (%)	56.1 ± 15.8 (52.9–59.3)	58.7 ± 16.2 (54.0–63.3)	53.4 ± 15.2 (48.9–57.8)	0.103	-
HR at AT (BPM)	171.0 ± 14.4 (168.1–173.9)	168.4 ± 14.9 (164.1–172.7)	173.8 ± 13.4 (169.8–177.8)	*0*.*013*	0.356^#^
HR at AT compared with the maximal HR (%)	91.5 ± 6.5 (90.2–92.8)	92.8 ± 11.2 (89.6–96.1)	91.4 ± 5.1 (89.8–92.9)	0.245	-
V̇O_2_ at AT (ml/kg/min)	38.3 ± 5.5 (37.2–39.4)	39.4 ± 5.3 (37.9–41.0)	37.1 ± 5.5 (35.5–38.7)	*0*.*036*	0.419^#^
V̇O_2_ at AT compared with the V̇O_2max_ (%)	86.5 ± 8.2 (84.8–88.1)	88.1 ± 7.7 (85.8–90.3)	84.8 ± 8.4 (82.3–87.3)	*0*.*049*	0.388^#^

Abbreviations: V̇O_2_, oxygen uptake; V̇O_2max_, maximal oxygen uptake; HR, Heart rate; AT, Anaerobic threshold; 95% CI, 95% confidence interval. Statistical analysis was carried out between the First and Second division referees.

^#^ small effect size,

^##^ medium effect size.

**Fig 1:**
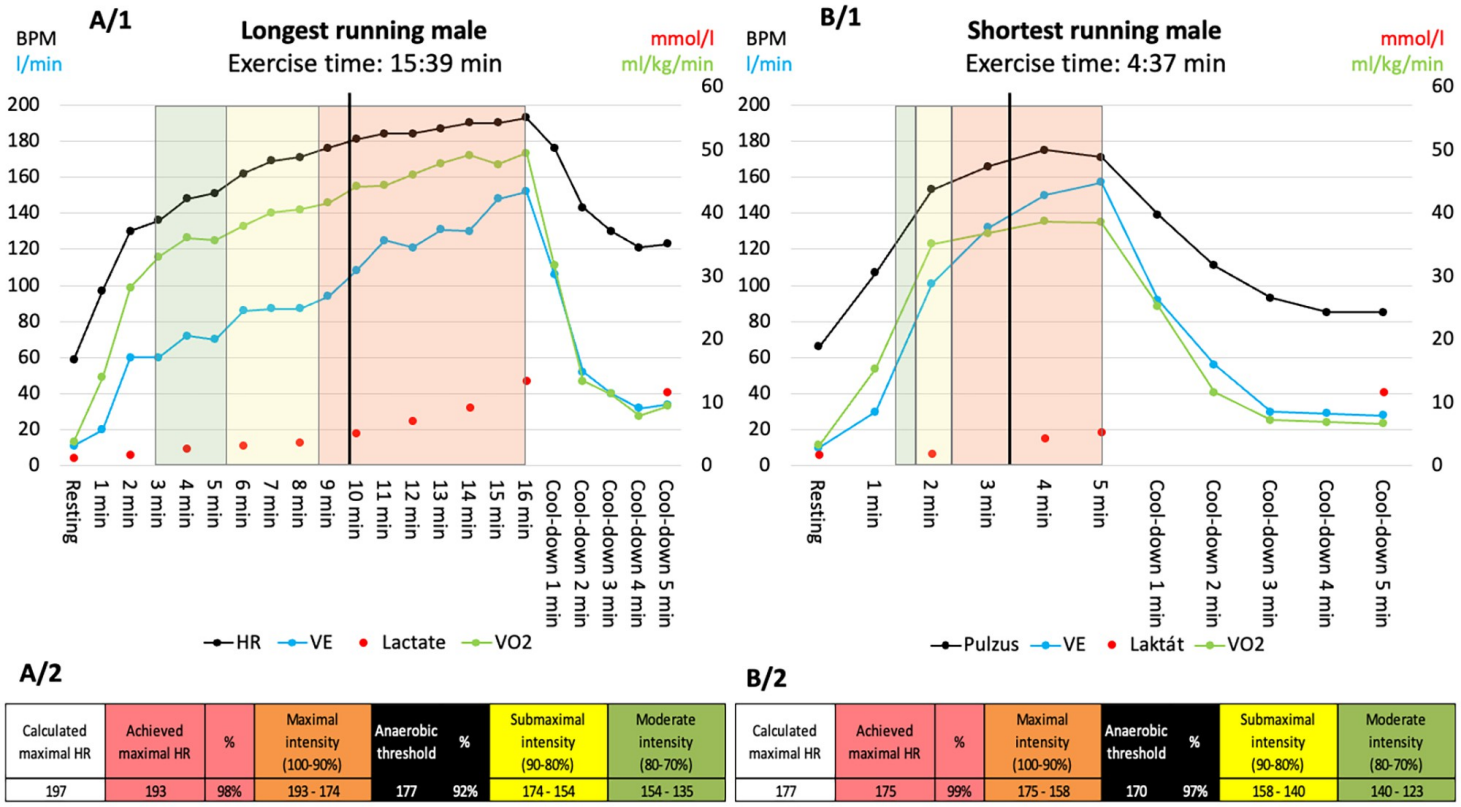
Representative cardiopulmonary exercise testing curves for referees. A/1 Cardiopulmonary exercise testing (CPET) results of a male referee who achieved the longest time in our sport-specific running protocol, B/1 CPET curves of a male referee who was among those who achieved the shortest running time. On the upper graphs, heart rate (HR, black line), ventilation (blue line), lactate values (red dots) and oxygen uptake (green line) are indicated. The vertical black line represents the anaerobic threshold, while the green, yellow, and red boxes indicate the HR intensity levels calculated from the achieved maximal HR (respectively, moderate, submaximal, and maximal intensity). Below, data on the A/2 and B/2 panels show the estimated maximal HR, the maximal achieved HR, the ratio of the maximal to the estimated HR, the maximal intensity HR zone, the HR at the anaerobic threshold, the ratio of the HR measured at the anaerobic threshold to the maximal HR, the submaximal HR zone, and the moderate intensity HR zone. Abbreviations: HR, Heart rate; VE, Ventilation; V̇O_2_, oxygen uptake.

**Fig 2:**
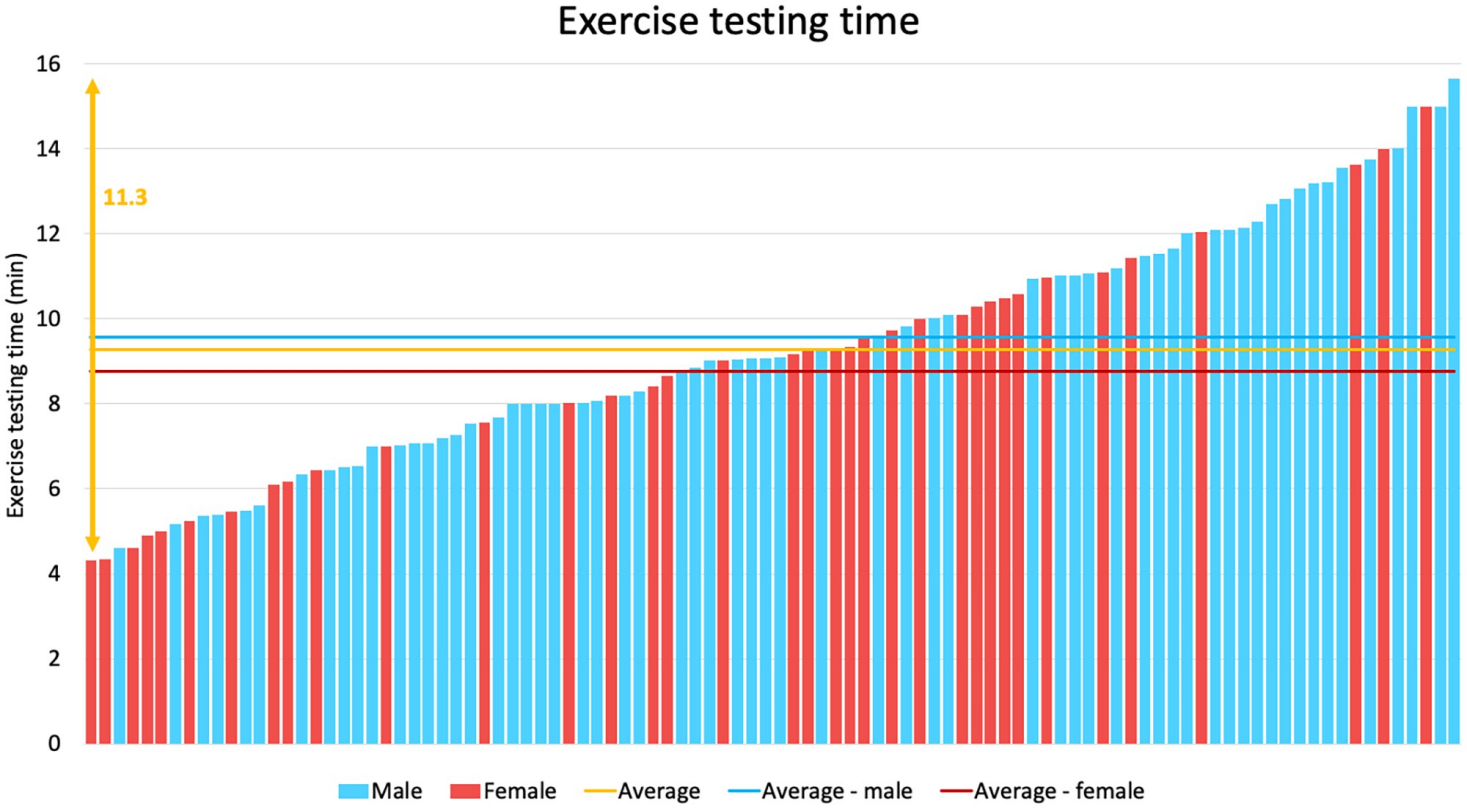
Exercise running times of elite Hungarian handball referees on a sport-specific running protocol. On the graph, each bar represents a referee. Male referees are shown with blue, female referees with red bars. The blue, red and yellow lines represent the average running times for male, female, and all referees, respectively. The yellow double arrow highlights the difference between the longest and shortest running times of the referees.

**Fig 3:**
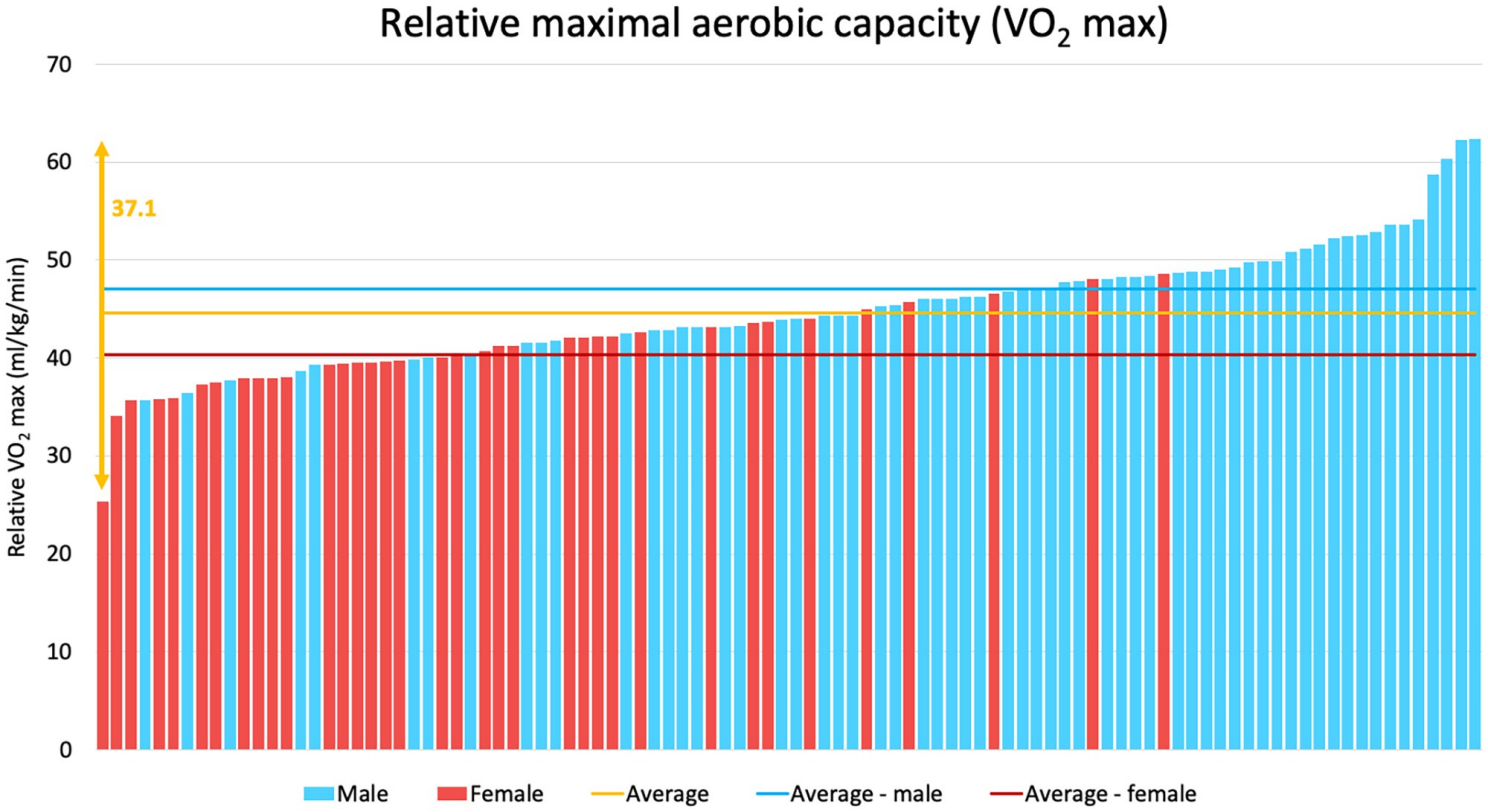
The relative maximal oxygen uptakes of elite Hungarian handball referees on a sport-specific running protocol. On the graph, each bar represents a referee. Male referees are shown with blue, female referees with red bars. The blue, red and yellow lines represent the average values for the male, female, and all referees, respectively. The yellow double arrow highlights the difference between the highest and lowest relative maximal oxygen uptake values of the referees.

In all the above-mentioned parameters, great individual differences could be observed among the referees, as presented on Fig 1. Exercise time ranged between 259.0 and 939.0 sec (Fig 2), V̇O_2max_ between 25.3 and 62.4 ml/kg/min (Fig 3), and anaerobic threshold time between 137.0 and 719.0 sec. In most of the cases, the measured values of V̇O_2max_ and ventilation overreached the estimated non-athlete reference values specified by the manufacturer.

### Correlations between body composition analysis results and fitness measurements

Considering the body composition analysis results and the fitness markers measured during the CPET examinations, positive correlations were found between the V̇O_2max_ values and height (r = 0.329, R^2^ = 0.108, p = 0.001) and FFMI (r = 0.456, R^2^ = 0.208, p < 0.001), while negative correlation was revealed with body fat percentage (r = -0.705, R^2^ = 0.497, p < 0.001). Correlating the maximal ventilation with body composition analysis parameters numerous relationships were found (height: r = 0.718, R^2^ = 0.516, p < 0.001; weight: r = 0.735, R^2^ = 0.540, p < 0.001; BMI: r = 0.556, R^2^ = 0.309, p < 0.001; FFMI: r = 0.734, R^2^ = 0.539, p < 0.001; body fat percentage: r = -0.461, R^2^ = 0.213, p < 0.001).

### Comparison of the first and second division referees

Compared to the first division referees, the second division referees reached higher exercise time, maximal HR and maximal ventilation values, with similar peak lactate and V̇O_2max_ values. The second division referees also reached their anaerobic threshold later. Data are shown in Table 2. About 9.8% of the first division referees and 14.3% of the second division referees did not reach the estimated values in ventilation, while 2.0% of the first and 4.1% of the second division referees did not reach the estimated values in oxygen uptake.

## Discussion

In our study, cardiorespiratory fitness measurements of elite handball referees were studied. Our main aims were i) to determine the fitness status of the Hungarian elite handball referees and to provide their CPET results for the first time in the literature. Since, in Hungary, there is no sex differentiation for referees in the refereed games, data were first analyzed collectively. By our results, the referees had a satisfactory fitness status compared to athletes who do mixed sports; however, remarkable individual differences were found. Our study lays grounds for further scientific research and training optimization of handball referees. We also aimed to carry out subgroup analyses ii) to compare the fitness status of referees working in the different divisions, and iii) to learn about the sex differences among elite referees. Surprisingly, the referees working in the second division achieved longer exercise time, supposedly because they were younger. Male and female referees reached similar exercise times on the CPET, however, differences in fitness status were observed.

According to previous literature data, handball referees perform at moderate intensity for most of the time during handball matches; however, in 3.6% of the game, they are exposed to heavy or severe load [24]. During these periods, they still need to be able to concentrate at maximum level. All the above underline the fact that referees need to prepare for the games with good physical condition, however, they perform trainings approximately 4 hours/week, while elite players train 10–20 hours/week [7]. Previous literature data suggest that higher physical load could influence referee decision making in a negative way in some sports, thereby proper physical preparation is necessary to prevent wrong calls [25].

According to our results, the measured oxygen uptake and ventilation values surpassed the estimated non-athlete reference values specified by the manufacturer in most of the cases. However, great individual differences were found in weekly training time, treadmill exercise time, aerobic capacity and anaerobic threshold time. These results highlight the necessity of routine physical fitness measurements, individual training programs, and reconsideration of minimal standards mandatory to be met.

The female referees achieved similar treadmill exercise times as the males, which is a welcome result, since they work in the same leagues. The referees from the second division achieved longer CPET examination times, anaerobic threshold times, and lower maximal ventilations. These results, as well as their higher HR, can be partially explained with their younger age [26].

To the best of our knowledge, this is the first study examining elite handball referees with cardiopulmonary exercise testing, thus, we could only compare these results with similar measurements of handball players and referees from other sports only. Comparing the results of this study with a report on 291 male elite German handball players, we can state that our referee group had lower maximal aerobic capacity and similar peak lactate levels [27].

Comparing the results of our younger referee population with the values of a small group of Brazilian handball referees, the maximal HR of referees was similar, while the maximal aerobic capacity proved to be lower in our study [24]. Also, compared with an older basketball referee population from Australia, the V̇O_2max_ was lower in our population [28]. The above-mentioned differences in maximal aerobic capacity can be explained by the fact that the other workgroups only used estimations to calculate the maximal aerobic capacity, while our team measured it directly by breath-by-breath measurement.

The anaerobic threshold was determined following the recommendations, through the complex analysis of several parameters measured during the CPET examinations. Different types of sports can highly influence the time spent in aerobic, mixed, and anaerobic phases during an incremental protocol CPET examination [29]. As commonly seen in team athletes, our referee population was in the anaerobic phase for nearly half of the CPET examinations, with relatively high HR and oxygen uptake at the anaerobic threshold. Compared to our results, the authors found lower HR in percentage to the achieved maximal HR, and lower oxygen uptake in percentage to the achieved V̇O_2max_ at the anaerobic threshold in a Turkish study conducted on handball players [30]. In another Croatian study among football players, higher percentages of HR and oxygen uptake were observed at the anaerobic threshold compared to our results [31].

In a Croatian study, no differences in maximal and average HR in referees between the two halves of a handball game were revealed [32]. These data support the fact that referees need to have a good long-lasting aerobic fitness level to referee the matches. In our population, we found a great difference between the referees with the best and the worst V̇O_2_ values, which could mean that some of the referees could meet the criteria to maintain a high-quality refereeing during the whole game, while those who achieved worse results during testing could have difficulties with their physical status during games.

In the current referee population, huge personal differences could be observed in the referees’ fitness status. However, better fitness status was not attached to refereeing in a higher division, possibly because previous experience and routine highly influences their assignments.

To better understand the fitness demands of handball referees, further examinations are required. Few studies are available where the physical load of the referees was measured during matches, but in those cases, no previous CPET examinations were carried out. Beyond the physical load during matches, the actual accuracy of refereeing should also be measured, and the correctness of their decisions should be correlated with the fatigue level of the referee at the time the calls are made.

With better training planning, improvements could be achieved in both aerobic and anaerobic fitness. As a result, match refereeing would be less physically demanding, resulting in better performance during the games. Individual training plans should be prescribed for the referees, containing both endurance and anaerobic components.

This is a single-center study conducted solely on Hungarian referees. The examined population should be broadened by extending the study to higher numbers of referees from different countries and ethnicities. A long-term follow-up should also be performed to evaluate their improvement and progress in their carrier. For a better understanding of the physical requirements of the handball referees, further examinations are required using data collected during matches, and comparisons should be performed between the results of CPET examinations and field measurements.

## Conclusion

In conclusion, our data underline the great individual differences among elite Hungarian handball referees, as well as the remarkable differences in the amount of training regularly done by them. Therefore, although refereeing experience highly affects the quality of refereeing, individual training plans should be prescribed for referees to reduce the differences observed in their physical fitness levels.

## Supporting information

S1 Dataset Minimal dataset of the examined referees. (XLSX)
